# Higher Prevalence and Age Susceptibility of Intracranial Aneurysm in Patients With Acoustic Neuroma

**DOI:** 10.3389/fneur.2020.591526

**Published:** 2020-11-02

**Authors:** Honghai You, Yue Bai, Ting Yu, Tiefa Zeng, Nan Huang, Wenzhong Mei, Changzhen Jiang, Dezhi Kang, Xiyue Wu, Fuxiang Chen

**Affiliations:** ^1^Department of Neurosurgery, The First Affiliated Hospital of Fujian Medical University, Fuzhou, China; ^2^Department of Ophthalmology, The First Affiliated Hospital of Fujian Medical University, Fuzhou, China; ^3^Department of Radiology, The First Affiliated Hospital of Fujian Medical University, Fuzhou, China

**Keywords:** acoustic neuroma, intracranial aneurysm, cerebrovascular angiography, risk factor, prevalence

## Abstract

**Introduction:** The purpose of this study was to verify whether the prevalence of intracranial aneurysm (IA) in patients with acoustic neuroma is greater than that in age- and sex-matched controls and to evaluate the independent risk factors related to the occurrence of IA.

**Methods:** We retrospectively analyzed 231 patients diagnosed with acoustic neuroma at our institute between 2015 and 2019 and 489 controls from the medical examination center. Cerebrovascular angiography was acquired from all subjects to assess the presence of IA or not. The prevalence of IA and risk factors associated with a higher IA occurrence were compared, respectively.

**Results:** Cerebral aneurysms were detected in 23 patients (10.0%) and 11 controls (2.2%). The prevalence of IA was significantly different between patients with acoustic neuroma and controls (*p* < 0.001), and the difference was mainly reflected in the age of 50 and above. In the subgroup analysis, there were distinct differences in several clinical features including age, hypertension, and tumor volume, and cystic change between patients coexisted with IA or not. However, age was a unique independent risk factor for coexistence of IA in patients with acoustic neuroma after multivariate logistic regression (OR 1.050, 95% CI 1.008–1.093, *p* = 0.019).

**Conclusions:** Our results demonstrate that patients with acoustic neuroma have a higher prevalence of IA than the general population. Older age is correlated with greater occurrence of IA in these patients.

## Introduction

Acoustic neuroma is the most common tumor in the cerebellopontine angle region, and surgical resection is the preferred treatment for most of the patients (1, 2). The prevalence of accidental unruptured intracranial aneurysm (IA) is estimated to be 2–3% among the general population (3) and increasing unruptured aneurysms being detected due to the advance and popularity of cerebrovascular angiography technologies (4). Accumulating evidence has uncovered that unexpected subarachnoid hemorrhage arising from aneurysm rupture during or after brain tumor resection is likely to deteriorate clinical outcomes and even results in death (5, 6). In recent years, the coexistence of brain tumors and IA is no longer a rare phenomenon and is getting more attention (7). However, the association between the acoustic neuroma and unruptured IA is still unclear.

Digital subtraction angiography (DSA) remains the gold standard for diagnosis of cerebral aneurysm. Additionally, CT angiography (CTA) and magnetic resonance angiography (MRA) have also been widely proven as reliable tools for aneurysm detection (8, 9). Previous researches have displayed that the prevalence of coexisting IA in patients with intracranial tumor ranges from 2.3 to 7.7% (5, 10), of which, meningioma and pituitary adenoma were considered as the most frequent brain tumors in relation to higher occurrence of cerebral aneurysm (10, 11), predominantly unruptured aneurysms. Clinical data have suggested that cerebellopontine region tumors are associated with a greater incidence of unruptured IA (12). Nevertheless, there is little study that has shed light on the prevalence of coexisting IA in patients suffering from acoustic neuroma. Besides, clinical characteristics or imaging features associated with the formation of IA are also poorly reported. Thus, we started such a retrospective study.

## Materials and Methods

### Participants

A total of 279 patients diagnosed with acoustic neuroma from the department of neurosurgery at our institute between January 2015 and December 2019 were retrospectively reviewed. Inclusion criteria included the following: (1) age range from 17 to 80 years old, (2) acquiring at least one cerebrovascular angiography imaging (CTA/MRA/DSA), (3) without a history of other brain tumor or brain surgery, (4) no evidence of neurofibromatosis or tuberous sclerosis, and (5) without a history of polycystic kidney disease and family history for aneurysms. In addition, age- and gender-matched controls (*n* = 489) came from the general population who went to our institute for a health screening in the same period. Cerebrovascular angiography estimation was performed in all controls with MRA, CTA, or DSA. The present study was approved, and the patient consent waived, by the local ethics committee of the First Affiliated Hospital of Fujian Medical University, and all methods were performed in accordance with the relevant guidelines and regulations.

### Clinical Characteristics and Imaging Evaluation of Acoustic Neuroma

The diagnosis of acoustic neuroma and its cystic change were mainly determined by preoperative brain MRI and intraoperative findings. Acoustic schwannoma volume was calculated by the maximal tumor diameter of the axial, coronal, and sagittal on contrast enhanced-MR images. In addition, acoustic neuromas were divided into left or right side according to its locations. Age and gender, as well as disease duration and tumor-initiated symptoms, were included together.

### Identification and Classifications of Intracranial Aneurysm

Determination of cerebral aneurysm was conducted together by two cerebrovascular neurosurgeons and two neuroradiological specialists in our institute. They carry out aneurysmal diagnosis or surgical treatment more than 300 cases each year. The clinical characteristics of all subjects were blind to four reviewers before diagnosis of aneurysm. Distributions of IA were classified into four types: internal carotid artery (ICA), anterior cerebral artery (ACA), middle cerebral artery (MCA), and vertebrobasilar artery (VBA). The size and number of cerebral aneurysms of each patient were also recorded. Patients with acoustic neuroma were separated into two groups according to their co-occurrence with unruptured aneurysm or not. Relative locations of them were classified as ipsilateral, contralateral, and bilateral (if multiple aneurysms were detected by individuals). Risk factors generally known as contributing to the formation of cerebral aneurysm, such as hypertension and smoking, were also collected.

### Statistical Analysis

The data were analyzed by SPSS 20.0 (SPSS Inc., Chicago, IL, USA). Continuous variables were compared using independent samples *t*-test. Chi-square-test or Fisher's exact test was used for analyses of gender, hypertension, side of tumor, and cystic change, as well as age-matched comparison of the incidence of IA between patients and controls. Factors associated with a higher incidence of IA in patients with acoustic neuroma at *p* < 0.10 on univariate analysis were further referred to unconditional multivariate logistic regression. A value of *p* < 0.05 was considered significant.

## Results

### Incidence of IA in Patients With Acoustic Neuroma and Age- and Gender-Matched Controls

A total of 231 cases of eligible unilateral acoustic neuroma were included in the analysis. Forty-six patients were excluded for absence of MRA, CTA, or DSA information, and the other two owing to definitive diagnosis of neurofibromatosis. Among them, removal of tumor was performed in 98.7% (228/231) of patients. Unruptured IAs were identified in 23 patients with acoustic neuroma (10.0%), whereas only 11 cases in the selected general population (2.2%, *p* < 0.001). There was no significant difference in age, gender, hypertension, or smoking between the groups. Detailed medical characteristics of the acoustic neuroma patients and controls are summarized in Table 1.

**Table 1:** Summary of clinical features in patients with acoustic neuroma and age- and gender-matched controls.

	**Acoustic neuroma**	**Controls**	***p*-value**
Age	51.4 ± 12.3	51.5 ± 11.6	0.964
Gender			0.066
Male	89 (38.5%)	224 (45.8%)	
Female	142 (61.5%)	265 (54.2%)	
Hypertension	47 (20.3%)	127 (26.0%)	0.100
Smoking	51 (22.1%)	134 (27.4%)	0.127
Aneurysm number	23 (10.0%)	11 (2.2%)	<0.001*
Single	18 (78.3%)	10 (100%)	
Double	5 (21.7%)	0 (0)	
Aneurysm size			NA
<5 mm	21 (91.3%)	11 (100%)	
≥5 mm	2 (8.7%)	0 (0)	
Aneurysm location			NA
ICA	17 (73.9%)	4 (36.4%)	
ACA	1 (4.3%)	4 (36.4%)	
MCA	2 (8.7%)	1 (9.1%)	
VBA	3 (13.0%)	2 (18.2%)	
Tumor volume (cm^3^)	19.73 ± 18.84	NA	NA
Tumor resection	228 (98.7%)	NA	NA
Relative location			NA
Ipsilateral	5 (21.7%)	NA	
Contralateral	13 (56.5%)	NA	
Bilateral	5 (21.7%)	NA	

*ICA, internal carotid artery; ACA, anterior cerebral artery; MCA, middle cerebral artery; VBA, vertebrobasilar artery; NA, not available*.

### Distributions of IA in Patients With Acoustic Neuroma and Controls

Single aneurysm was seen in 18 patients (78.3%) and 11 controls (100%), and double aneurysms (21.7%) in 5 patients. Most of them were smaller than 5 mm, with a proportion of 91.3 and 100% in each group, respectively. The locations of aneurysm were involved in ICA, ACA, MCA, and VBA. Anterior circulation aneurysms account for over 80% in both groups (87.0 and 81.8%, respectively). In addition, we further divided all subjects into four categories according to their age (<40, 40–49, 50–59, and ≥60). The incidence of IA in patients with acoustic neuroma was 12.1 and 15.9% in the 50- and 60-year-old subgroups, in which it was 4.2 and 0.8% in the controls, both with significant differences (*p* = 0.041 and <0.001). Details can be referred to Tables 1, 2.

**Table 2:** Age-matched comparison of the incidence of intracranial aneurysm in patients with acoustic neuroma and controls.

**Ages**	**Patients with acoustic neuroma**			**Controls**			***p*-value**
	**Number**	**Coexisting IA**	**Prevalence (%)**	**Number**	**Coexisting IA**	**Prevalence (%)**	
<40	38	3	7.9	62	2	3.2	0.365
40–49	64	2	3.1	159	2	1.3	0.325
50–59	66	8	12.1	143	6	4.2	0.041*
≥60	63	10	15.9	125	1	0.8	<0.001*

*IA, intracranial aneurysm*.

### Risk Factors of Coexisting IA in Patients With Acoustic Neuroma

To explore the risk factor correlation with higher incidence of coexisting aneurysm in patients with acoustic neuroma, all 231 patients were further divided into two subgroups. Group I (*n* = 23) was patients with coexistence of acoustic neuroma and cerebral aneurysm, whereas group II (*n* = 208) was acoustic neuroma alone.

The mean age of group I was 57.2 ± 11.1 years old, whereas group II was 50.8 ± 12.3 (*p* = 0.017). Tumor mean volume of group I was calculated to be 14.83 ± 10.63 cm^3^, and 20.27 ± 19.44 cm^3^ in group II (*p* = 0.042). We also evaluated the cystic change of acoustic schwannoma, with a positive occurrence of 87.0% in group I and 65.4% in group II (*p* = 0.036). Significant differences of the abovementioned three parameters between the groups were found, suggesting that they might be potential risk factors for the co-occurrence of IA in patients with acoustic schwannoma. Patients most frequently presented with symptoms of brain tumor in both groups (*p* = 1). Comparisons of other clinical features, including disease duration, hypertension, smoking, and tumor side, were also performed. However, there was no significant difference in these four indicators (all *p* > 0.05). Demographic and clinical features of two subgroups of acoustic neuroma are presented in Table 3.

**Table 3:** Comparison of clinical characteristics in patients with acoustic neuroma that coexisted with intracranial aneurysm or not.

	**Group I**	**Group II**	***p*-value**
Age	57.2 ± 11.1	50.8 ± 12.3	0.017*
Gender			0.950
Male	9 (39.1%)	80 (38.5%)	
Female	14 (60.9%)	128 (61.5%)	
Hypertension	8 (34.8%)	39 (18.8%)	0.097
Smoking	6 (26.1%)	45 (21.6%)	0.625
Tumor onset	23 (100.0%)	206 (99.0%)	1.000
Disease duration	33.87 ± 40.80	41.87 ± 66.68	0.574
Tumor side			0.282
Left	102 (49.0%)	106 (51.0%)	
Right	14 (60.9%)	9 (39.1%)	
Tumor volume (cm^3^)	14.83 ± 10.63	20.27 ± 19.44	0.042*
Cystic change	20 (87.0%)	136 (65.4%)	0.036*

The age, tumor volume, and cystic change of acoustic neuroma were further referred to multivariate logistic regression analysis based on comparative results between patients coexisted with brain aneurysm or not. We also included hypertension, with an increased tendency (*p* < 0.10) for IA formation in this analysis. The result indicated that only patient's age was correlated with a higher incidence of IA in patients with acoustic neuroma (*p* = 0.019), with odds ratio (OR 1.050) and 95% confidence interval (95% CI 1.008–1.093). Statistical information can be referred to Table 4.

**Table 4:** Independent risk factor correlated with a higher incidence of intracranial aneurysm in patients with acoustic neuroma.

**Parameter**	**OR**	**95% CI**	***p*value**
Age	1.050	1.008–1.093	0.019*

*OR, odds ratio; CI, confidence interval*.

## Discussion

The overall incidence of cerebral aneurysm in patients with brain tumor was less than 1% decades ago (13). However, increasing rate of unruptured IA is detected due to the widespread utility of cerebrovascular diagnostic manners including CTA, MRA, and DSA (14, 15). The prevalence of IA in patients diagnosed with pituitary adenoma or meningioma was reported to be 2.3 and 7.7% in recent studies, respectively (5, 10). To the best of our knowledge, the present study first discovered that the coexistence of cerebral aneurysm in patients suffering from acoustic neuroma is even up to 10%. There was a significant difference compared with the age- and gender-matched controls from the routine physical examination population, suggesting a higher occurrence of IA in patients with acoustic neuroma relative to the general population.

Several possible mechanisms have been proposed for interpretation of an increased prevalence of cerebral aneurysm in patients with diagnosis of brain tumors (5, 10, 12). In response to chronic hypertension caused by persistent mass effect of brain tumor on adjacent tissues, mean arterial blood pressure increases correspondingly to maintain adequate cerebral perfusion pressure. Long-term compensatory elevation of arterial blood pressure leads to hemodynamic unbalance, especially the bifurcation locations of cerebral artery, which has been widely accepted as a pathological process, increasing susceptibility to formation even rupture of aneurysms (16, 17). This hypothesis is supported by the fact that tumor volume has been shown to play an important role in the formation of cerebral aneurysm (5). Hormone levels and types were also considered to play a pivotal role in the coexistence of brain tumor and aneurysm in previously reported researches. For example, growth hormone-secreting pituitary adenoma has been frequently reported as responsible for an elevated coexistence rate of cerebral aneurysm (10, 18). In addition, decline level of estrogen in perimenopausal women was also contributing to a rising risk of IA pathogenesis (19). Besides, tumor cell infiltration into vascular wall and genetic variations also play a considerable role in the formation of aneurysms (5, 20).

However, to date, the exact mechanism of a higher incidence of IA in patients with acoustic neuroma remains unclear. Subarachnoid hemorrhage due to rupture of ipsilateral pseudoaneurysm was found in patients with acoustic schwannoma who underwent stereotactic irradiation (21, 22), indicating that radiation-induced vascular injury probably facilitated the formation of aneurysms (23). However, patients with acoustic neuroma included in the present study had an absence of any radiative history of brain tumors. Similarly, none of the patients were suffering from polycystic kidney disease that has been demonstrated to be linked with a higher prevalence of unruptured IA (4). Unquestionably, a possibility should be kept in mind that the coexistence of acoustic neuroma and IA may be a coincidence.

On the basis of the above results, the author attempted to reveal the independent risk factors of coexisting IA in acoustic neuroma patients. As a result, older age was correlated with a heavy incidence of unruptured IA in patients with acoustic neuroma. Coincidentally, the parallel concept has also been presented in a similar study working on assessing factors associated with cerebral aneurysm formation in patients with pathological confirmed pituitary adenoma (10). On the other hand, it is well-known that cerebral arteriosclerosis is common in elderly people and that the degree of sclerosis increases with age. Part of the literature concerning the association between arteriosclerosis and cerebral aneurysm supports our results as well, in which cerebral arteriosclerosis may predispose to aneurysm formation or even cause aneurysm rupture (12, 24).

Aneurysmal subarachnoid hemorrhage has an awful mortality and disability rate. The accidental finding of aneurysms during brain tumor resection will undoubtedly increase the mental burden of neurosurgeons and even lead to poor clinical outcomes (13, 25). Although the treatment of occasional IAs is still controversial, it is necessary to identify whether a cerebral aneurysm coexisted in patients with brain tumor preoperatively, which might be likely to improve safety in the perioperative period. High diagnostic accuracy of MRA, CTA, and DSA on IA has been confirmed in a variety of researches. Therefore, the present study did not further explore the diagnostic sensitivity of these methods. Given the higher incidence of cerebral aneurysms in patients diagnosed with acoustic neuroma, the authors believe that it is meaningful to carry out at least a kind of cerebrovascular angiography before removal of acoustic neuroma, especially in elderly patients.

There are several limitations in the present study. First, almost all patients with acoustic neuroma undergo CTA testing before surgery, partly for the purpose of understanding clearly tumor blood supply. For subjects from the healthy testing general population, they preferred to select a noninvasive MRA, but technically, both MRA and CTA have been demonstrated to be sensitive enough for detection of brain aneurysms. Second, there may be a possibility of selective bias in our study, although the incidental rate of aneurysms in the control group is similar to previous studies. Therefore, prospective studies or population-based series should be conducted in the future to present more robust evidence. Third, although we have performed a follow-up on the neurological function in patients with acoustic neuroma, the dynamic assessment of coexisting aneurysm is clearly insufficient, which limits us to further explore the potential mechanism of higher occurrence of cerebral aneurysm.

## Conclusions

Prevalence of IA in patients with acoustic neuroma is higher than that in age- and sex-matched controls. Elderly patients with acoustic neuroma are susceptible to coexistence with cerebral aneurysm. For these patients, preoperative cerebrovascular angiography may be necessary.

## Data Availability Statement

The raw data supporting the conclusions of this article will be made available by the authors, without undue reservation.

## Ethics Statement

The studies involving human participants were reviewed and approved by Ethics Committee of the First Affiliated Hospital of Fujian Medical University. The ethics committee waived the requirement of written informed consent for participation.

## Author Contributions

HY and YB drafted the manuscript. TY, TZ, NH, DK, and WM participated in the literature collection. NH, CJ, and DK analyzed the MRI features. XW and FC reviewed and revised the manuscript. All authors read and approved the final manuscript.

## Conflict of Interest

The authors declare that the research was conducted in the absence of any commercial or financial relationships that could be construed as a potential conflict of interest.
